# Comparative safety of infliximab and adalimumab on pregnancy outcomes of women with inflammatory bowel diseases: a systematic review & meta-analysis

**DOI:** 10.1186/s12884-022-05191-z

**Published:** 2022-11-19

**Authors:** Han Wang, Yue Hu, Fang Chen, Mengdie Shen

**Affiliations:** 1 Department of gynecology and obstetrics, Women’s Hospital, School of Medicine, Zhejiang University, 310003 Hangzhou, Zhejiang Province P.R. China; 2 Department of Gastroenterology, First Affiliated Hospital of Zhejiang Chinese Medical University, 310006 Hangzhou, Zhejiang Province P.R. China; 3 Department of Gastroenterology, Zhejiang Hospital of Integrated Traditional Chinese and Western Medicine, 310006 Hangzhou, Zhejiang Province P.R. China; 4 Department of Internal Medicine, Women’s Hospital, School of Medicine, Zhejiang University, 310003 Hangzhou, Zhejiang Province P.R. China

**Keywords:** Inflammatory bowel disease, Adverse pregnancy outcomes, Infliximab, Adalimumab

## Abstract

**Background:**

Inflammatory bowel disease (IBD) is a condition that affects most of the digestive tract. There is no report of fertility reduction in medically managed IBD women compared with the general population. On the other hand, active IBD can lead to significantly decreased fertility. Over the previous 2 decades, anti-tumor necrosis factor (anti-TNF) has been an effective treatment for managing patients with IBD, increasing the use of infliximab and adalimumab in clinical practice. However, it is unclear which biologics are better for pregnant women with IBD.

**Aim:**

We conducted a systematic review and meta-analysis for the risk of adverse pregnancy outcomes following treatment with infliximab and adalimumab in women with IBD.

**Methods:**

Bibliographic databases were retrieved from their inception to July 2022. The results were adverse pregnancy outcomes, including congenital malformations and spontaneous abortion.

**Results:**

A total of 8 studies included 527 pregnant women with IBD. Of these, 343 received infliximab, and 184 received adalimumab therapy. Compared to adalimumab, adverse pregnancy outcomes were not increased in infliximab therapy including congenital malformations and spontaneous abortion.

**Conclusion:**

Infliximab and adalimumab therapy did not show the difference of risk in adverse pregnancy outcomes in women with IBD.

**Systematic review registration:**

http://www.crd.york.ac.uk/PROSPERO, identifier: CRD 42,021,277,869.

## Introduction

Inflammatory bowel disease (IBD) is a chronic inflammatory disease that affects most of the digestive tract [1]. According to the phenotypic manifestations, IBD can be divided into ulcerative colitis (UC) and Crohn’s disease (CD). IBD affects people of all ages, including young patients in the reproductive stage. Active disease is associated with an increased risk of adverse pregnancy outcomes (APOs) such as preterm delivery, low birth weight, spontaneous abortion and congenital malformations[2]. However, the cause of IBD is still unknown, but there is increasing evidence of familial susceptibility to transmittable intestinal antigens [3]. The peak age of IBD diagnosis occurs during the childbearing years. Therefore, treatment of IBD during pregnancy is very common [4]. However, the probable adverse effects of drugs on an unborn infant, complications after different delivery modes, lactation, predisposal to genetic diseases, and other beliefs may lead to intentional failure to have children [5].

Tumor necrosis factor (TNF)-α is a major proinflammatory and pathological cytokine having pleiotropic effects on various cell types [6]. Its plays a key role in the pathogenesis of systemic inflammatory diseases such as rheumatoid arthritis and IBD. Anti-TNF-α therapy was the first type of biotherapy approved to treat IBD, which revolutionized IBD treatment [7, 8]. At present, infliximab and adalimumab are the most widely used in clinical practice for IBD treatment. When compared with placebo, infliximab and adalimumab demonstrate similar clinical outcomes [7, 9–12], including the requirements for corticosteroids, rates of remission, disease-related surgery, and hospitalizations. Of note, data on pregnant women are limited, and the differences between the study populations preclude determining comparative APOs. Nevertheless, there is growing recognition of the need for studies on the comparative APOs for pregnant women to inform clinical practice accurately. Our previous study has shown that anti-TNF can be advocated for IBD women with pregnancy. With this current meta-analysis, our study aims to quantify the risk of APOs in IBD women exposed to infliximab and adalimumab. The outcome of this study will provide valuable evidence for guiding the best clinical decision-making.

## Method

The systematic evaluation was conducted using predefined protocols and reported according to the preferred reporting items of the system evaluation and the presentation of the system evaluation meta-analysis (PRISMA) incorporating health care interventions (PROSPERO registration number: CRD 42,021,277,869).

### Search strategy

Medline, PubMed, Web of Science, Embase, and Cochrane Library were searched to identify relevant studies assessing the pregnancy outcomes in women with IBD who received infliximab or adalimumab at pregnancy. Only studies published in English were included. At the same time, we searched the reference list of the retrieved articles to carry out other relevant research as completely as possible. The database search was performed on 11th September 2021, and then updated on 12th July 2022.

### Study selection

Two reviewers independently reviewed the title and summary of each article to eliminate duplicates, annotations, case reports, and small case series (*n* < 10). We screened the titles and abstracts of published articles and excluded studies unrelated to this study. Full-text articles were obtained if at least one reviewer considered them qualified. Our analysis included RCTs, observational studies, and case-control studies. Infliximab and adalimumab received marketing authorization from the US Food and Drug Administration, or the European Medicines Agency were also considered in our research. In literature selection, any differences were resolved through discussion and consultation.

Inclusion criteria: (1) Patients: Pregnancy in IBD patients older than 18 years who were taking infliximab or adalimumab; (2) Intervention: infliximab therapy at any stage of pregnancy; (3) Comparator: adalimumab therapy at any point during pregnancy; (4) Outcomes: the primary outcome was adverse pregnancy outcomes in patients with IBD pregnancy, including preterm birth, low birth weight, spontaneous abortion, and congenital malformations.

Exclusion criteria: (1) trials evaluating any medical treatment protocol other than infliximab and adalimumab; (2) studies on the use of infliximab or adalimumab in pregnant women for an underlying disease other than IBD; (3) inadequate or absent control groups, information on birth outcomes was not available in full, provision of data obtained from other research, or trials assessing differences between combination therapy and monotherapy only.

### Data extraction and quality assessment

Two reviewers reviewed the full-text candidate articles to confirm the characteristics of the target population, disease treatment, medications used, the number of sample populations, and adverse results. The article’s authors were contacted if the data were not available fully. Any dispute was settled through mutual discussion or negotiation with the third reviewer.

The Newcastle Ottawa scale (NOS) was selected for assessing the literature qualities of selected case-control and cohort studies [13]. NOS is an assessment tool for the quality of observational research. It classified quality levels into three categories: group comparability, study group selection, exposure (case-control study), or result (cohort study). The research with 5 or more points in the 9-point system was rated as high-quality, while other studies were considered low-quality. Any differences between reviewers were discussed and resolved by negotiation.

### Statistical analysis

We used the Mantel Haenszel method to calculate the odds ratio (OR) and 95% confidence interval (CI). The selection of a random or fixed model was based on heterogeneity analysis [14]. *I*^*2*^ statistics were used to evaluate heterogeneity, and a fixed-effect model was used for *I*^*2*^ < 50%, while a random-effect model was used for *I*^*2*^ ≥ 50% [15]. *I*^*2*^ > 50% indicated significant heterogeneity in the study. For the evaluation of publication bias, we checked the asymmetry of the funnel chart, which was more conducive to determining whether small studies were effective [16]. All statistical analyses were performed using RevMan (version 5.3.0, Copenhagen, Denmark).

## Results

### Study selection

**Fig. 1:**
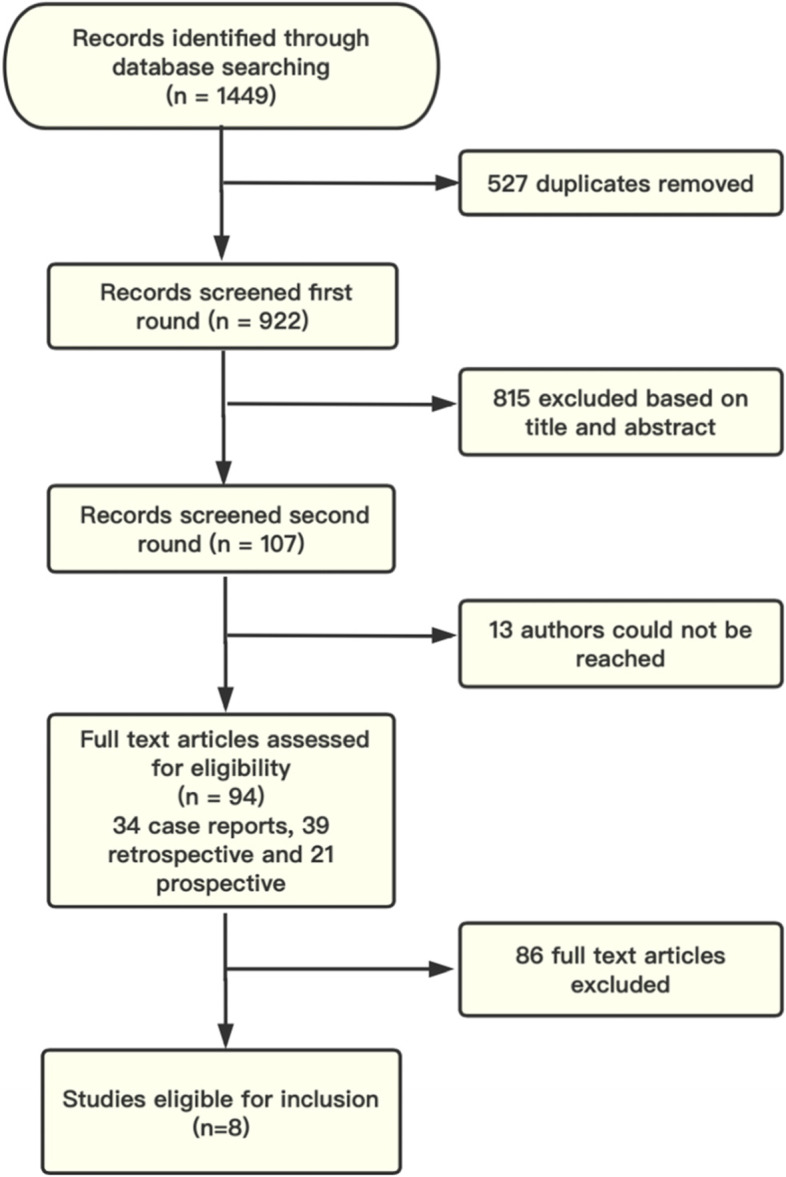
Study flow diagram. Records were identified through database searches and grey literature. A total of 94 articles met the criteria for full-text review, and 8 of them were finally included in the meta-analysis

The initial electronic and manual searches produced a total of 1449 studies. Among them, 94 studies met our research criteria, including 34 reports or small case series (n<10), 39 retrospective studies, and 21 prospective studies. Finally, 8 studies (4 prospective studies, 2 retrospective studies, and 2 both) with final compliance for selection criteria were included in the meta-analysis (Fig. 1).

### Study characteristics

A total of 527 cases were included in the 8 eligible studies. In addition, 343 pregnant women with IBD were treated with infliximab therapy and 184 with adalimumab therapy. These studies characteristics and pregnancy outcomes are summarized in Tables 1 and 2, respectively.

**Table 1:** Characteristics of the studies included on the use of Infliximab and Adalimumab during pregnancy

Study	Design	Pregnancies (n)	No. of pregnancies in infliximab-exposed group	No. of pregnancies in adalimumab-exposed group	Study quality
Kiely CJ [17]	P	21	10 Infliximab	11 Adalimumab	☆☆☆☆☆☆
Schnitzler F [18]	P	42	35 Infliximab	7 Adalimumab	☆☆☆☆☆☆☆
Seirafi M [19]	P + R	128	86 Infliximab	42 Adalimumab	☆☆☆☆☆☆☆
Casanova MJ [20]	R	29	20 Infliximab	9 Adalimumab	☆☆☆☆☆☆☆
Julsgaard M [21]	P	80	44 Infliximab	36 Adalimumab	☆☆☆☆☆☆
Arsenescu R [22]	R	10	8 Infliximab	2 Adalimumab	☆☆☆☆☆
Slama W [23]	P	186	122 Infliximab	64 Adalimumab	☆☆☆☆☆
Zelinkova Z [24]	P + R	31	18 Infliximab	13 Adalimumab	☆☆☆☆☆☆

*R* Retrospective, *P* Prospective

**Table 2:** Pregnancy outcomes (proportion of adverse outcome to number of exposed pregnancies) of included studies

Outcomes Group	preterm delivery		low birth weight		spontaneous abortion		congenital malformations	
	A	B	A	B	A	B	A	B
Kiely CJ [17]	2/21		2/21		0/10	1/11^a^	0/21	
Schnitzler F [18]	8/42		6/42		6/35	1/7	0/35	1/7
Seirafi M [19]							1/86	0/42
Casanova MJ [20]							0/20	1/9
Julsgaard M [21]	3/80		6/80				2/44	1/36
Arsenescu R [22]					1/8	0/2	0/10	
Slama W [23]					1/122	1/64	3/122	1/64
Zelinkova Z [24]					1/18	2/13	1/18	0/13

A: exposed to Infliximab B: exposed to Adalimumab

^a^This patient underwent an emergency colectomy following failed treatment with adalimumab. The stillbirth occurred at week 21 gestation, 11 days following the colectomy

### Adverse pregnancy outcomes

Eight studies reported the APOs associated with spontaneous abortion and congenital malformations after exposure to biological agents in pregnant women with IBD. The OR for the pooled crude rates of APOs was 0.74 (95% CI: 0.33, 1.66; *P* = 0.46), comparing infliximab (*n* = 343) with adalimumab (*n* = 184), without obvious heterogeneity (*P* = 0.95, *I*^*2*^ = 0%).

#### Spontaneous abortion

Five studies reported the outcome of spontaneous abortion in IBD pregnant women exposed to biological agents. The pooled OR for the crude rate of spontaneous abortion was 0.61 (95% CI: 0.19, 1.97; *P* = 0.41). There was no heterogeneity between studies when comparing infliximab (*n* = 193) and adalimumab (*n* = 97) (*P* = 0.93, *I*^*2*^ = 0%).

#### Congenital malformations

**Fig. 2:**
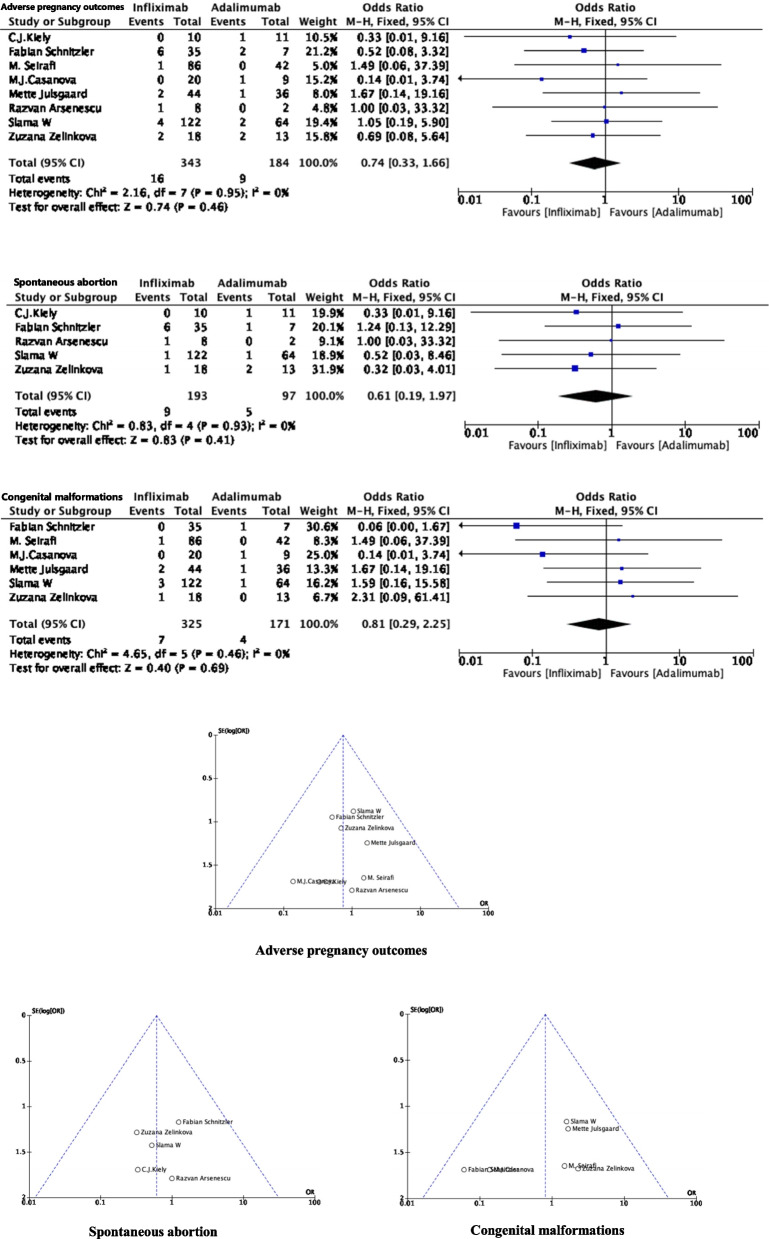
Risk of APOs in pregnant women treated with infliximab and adalimumab for IBD.

Six studies reported congenital malformation outcomes in IBD pregnant women exposed to anti TNF-α. The pooled OR for the crude rate of congenital malformations was 0.81 (95% CI: 0.29, 2.25; *P* = 0.69) comparing patients treated with infliximab (*n* = 325) and adalimumab (*n* = 171). There was no significant heterogeneity observed between studies (*P* = 0.46, *I*^*2*^ = 0%) (Fig. 2).

## Sensitivity analysis

The sensitivity analysis with “leave-one-out " shows that our results were robust. In addition, we excluded each included study separately and found that the original research results did not change substantially.

Among the 8 studies included, there was one with a large number of patients, so we removed this study [23] and evaluated again. Our results are as follow:

Seven studies reported the APOs associated with spontaneous abortion and congenital malformations after exposure to biological agents in pregnant women with IBD. The OR for the pooled crude rates of APOs was 0.66 (95% CI: 0.26, 1.68; *P* = 0.38), comparing infliximab (*n* = 221) with adalimumab (*n* = 120), without obvious heterogeneity (*P* = 0.92, *I*^*2*^ = 0%).

### Spontaneous abortion

Four studies reported the outcome of spontaneous abortion in IBD pregnant women exposed to biological agents. The pooled OR for the crude rate of spontaneous abortion was 0.63 (95% CI: 0.17, 2.29; *P* = 0.48). There was no heterogeneity between studies when comparing infliximab (*n* = 71) and adalimumab (*n* = 33) (*P* = 0.85, *I*^*2*^ = 0%).

### Congenital malformations

**Fig. 3:**
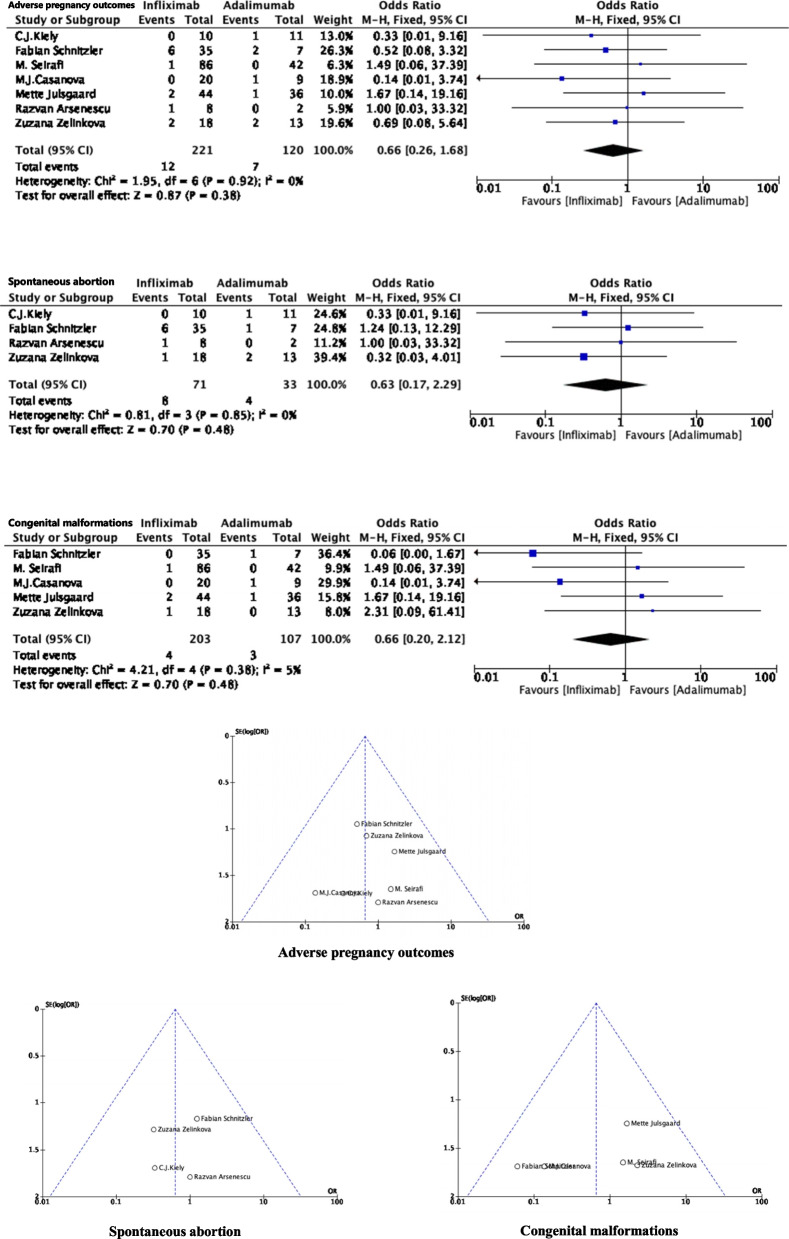
Risk of APOs in pregnant women treated with infliximab and adalimumab for IBD (sensitivity analysis)

Five studies reported congenital malformation outcomes in IBD pregnant women exposed to anti TNF-α. The pooled OR for the crude rate of congenital malformations was 0.66 (95% CI: 0.20, 2.12; *P* = 0.48) comparing patients treated with infliximab (*n* = 203) and adalimumab (*n* = 107). There was no significant heterogeneity observed between studies (*P* = 0.38, *I*^*2*^ = 5%) (Fig. 3).

## Discussion

The high incidence rate of IBD accounts for more than 0.3% of cases in North America and many European countries. Many patients are diagnosed with IBD as young adults, which affects the peak of fertility and family planning [25]. Many patients take 5-amino salicylate (5-ASA), corticosteroids, biologics, and immunosuppressants [26, 27]. Female patients with active IBD have an ascendant risk of developing adverse maternal and infant outcomes. Therefore, the best practice for both mothers and infants is to optimize disease control during pregnancy [28, 29]. However, before or during pregnancy, women often reduce or stop prescribing drugs without discussing them with their physicians [30]. Most patients with IBD require long-term medication to control and maintain the disease [31, 32]. All anti-TNF drugs effectively maintain clinical remission and mucosal healing in the case of infliximab and adalimumab therapy [33]. The Toronto [28] and ECCO [4, 32] consensus statements showed that sustained remission is important for pregnancy success. At the same time, anti-TNF therapy does not lead to adverse maternal and infant outcomes. For 5-ASA, thiopurines, or anti-TNF-α, women with well-controlled medical maintenance therapy should continue treatment throughout pregnancy. Nevertheless, patient misconceptions and unsubstantiated fears of treatment teratogenicity contribute to medication non-adherence during pregnancy and breastfeeding [34–38]. Anti-TNF therapies are reported to be effective in many high-quality placebo-controlled trials, but data comparing the APOs of the two drugs in clinical practice are limited. Women with IBD already in the active phase before pregnancy have a higher risk of premature birth. This is also associated with poorer fetal outcomes, including the ascendant risk of small gestational age preterm delivery and low birth weight [39]. Therefore, it is necessary to study the comparative APOs of therapies for pregnant women with IBD. The basis for selecting anti-TNF monoclonal antibodies is not clearly stated in the consensus. Moreover, there is no evidence on the comparative APOs of infliximab and adalimumab in pregnant women with IBD.

Although many regulatory bodies strongly encourage various studies to incorporate pregnant women and women of childbearing age in RCTs, pregnant and lactating women are often excluded from the trial due to unknown potential harm to the fetus. Therefore, treatment options during pregnancy often lack strong evidence-based recommendations. Safety information comes from voluntary reports of adverse events or uncontrolled observational studies during post-marketing monitoring. The current meta-analysis explored the risk of adverse pregnancy outcomes (we collected the data on preterm delivery, low birth weight, spontaneous abortion, and congenital malformations) following the infliximab and adalimumab therapy in women with IBD. 343 pregnant women with IBD who received infliximab were compared with 184 pregnant women who received adalimumab. In our study, the ORs for adverse pregnancy outcomes in IBD patients taking infliximab therapy during pregnancy compared with those taking adalimumab were 0.74 (95% CI: 0.33, 1.66). The OR of pooled crude rates of congenital malformations and spontaneous abortion were 0.81 (95% CI: 0.29, 2.25) and 0.61 (95% CI: 0.19, 1.97), respectively. However, no significant difference was observed between infliximab and adalimumab in the risk of APOs.

**Fig. 4:**
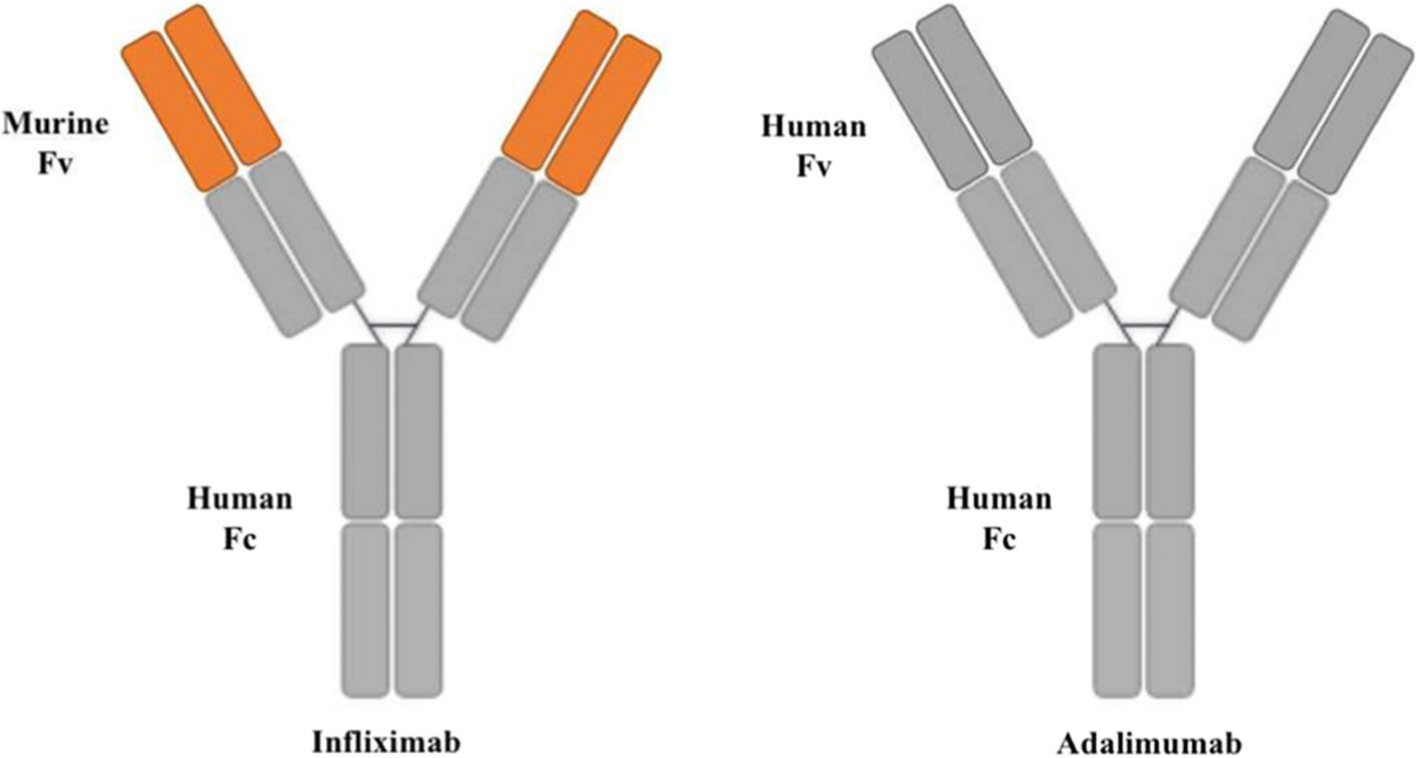
Simplified diagrams of the molecular structures of two TNF antagonists

A retrospective analysis of “real” data from 3205 patients showed that infliximab was superior to adalimumab and certolizumab in treating CD [40]. In contrast, a series of recent studies in Austria showed that infliximab and adalimumab were equally effective in treating CD [41]. These results were neither uniform nor different. During pregnancy, the pharmacodynamics and pharmacokinetics of many drugs changes. Infliximab and adalimumab are complete IgG1 anti-TNF monoclonal antibodies with strong anti-inflammatory effects. They are actively transferred through the placenta in exponential form through the Fc receptor from the second trimester of pregnancy. In one small study by Seow et al [42], 15 pregnant women treated with infliximab and 10 pregnant women treated with adalimumab increased infliximab levels while adalimumab levels remained stable. IgG1 is transported across the placenta more readily than other IgG subclasses, with passage increasing exponentially toward the later stages of pregnancy [43]. Several cohort studies, systematic reviews, and meta-analyses have reported no adverse effects of infliximab, adalimumab, or certolizumab during pregnancy. There was no increased risk of congenital malformations, preterm birth, spontaneous abortion, or low birth weight [20, 44–50]. These observations were consistent with our study, which shows that pregnant women with IBD on infliximab and adalimumab therapy did not show the difference in the risk of APOs. Like other anti-TNFs, infliximab and adalimumab are classified as pregnancy category B (no documented human toxicity) by the US Food and Drug Administration. Infliximab is a murine/human chimeric anti-TNF- α Monoclonal antibody containing murine variable and human IgG1 constant regions. Infliximab contains 25% murine sequences, which may be related to the occurrence of adverse reactions. Adalimumab is a completely humanized anti-TNF- α Monoclonal antibody with no difference from normal human IgG1 [6, 51] (Fig. 4). Infliximab and adalimumab can efficiently cross the placenta in the second and third trimesters due to their specific FcRn receptor-mediated mechanisms [43, 52].

Our study had some limitations. First, the conclusions of the expert group were based on the opinions of experts and the interpretation of existing evidence. However, the evidence for certain scenarios included in the study was limited. We attempted to obtain the data on preterm delivery and low birth weight. However, some articles did not clearly distinguish between the infliximab and adalimumab groups. Thus we analyzed the risk of congenital malformations and spontaneous abortion. Secondly, our results did not rule out other factors, such as patient compliance, local drug access, or lack through the local medical level. Inevitably, clinicians need to consider the best treatment for a particular patient, considering the numerous associated factors. Although good control of disease activity during pregnancy is beneficial for a better outcome, there is no strong evidence to prove the safety of drug exposure to developing fetuses.

In conclusion, clinicians should be very cautious. There are still many aspects that need optimization in this field. Thus, larger prospective trials involving pregnant patients are required to establish a drug safety database. Additionally, larger RCTs research outcomes will help promote the clinical decision-making of the treatment of women with IBD of childbearing age to optimize the pregnancy outcomes.

## Data Availability

The original contributions presented in the study are included in the article/supplementary material, further inquiries can be directed to the corresponding author/s.

## References

[1] Torres J, Mehandru S, Colombel JF, Peyrin-Biroulet L (2017). Crohn’s disease. Lancet.

[2] Practice CoO (2019). Quantitative Blood Loss in Obstetric Hemorrhage: ACOG COMMITTEE OPINION, Number 794. Obstet Gynecol.

[3] Keighley MR, Stockbrugger RW (2003). Inflammatory bowel disease. Aliment Pharmacol Ther.

[4] Gionchetti P, Dignass A, Danese S, Magro Dias FJ, Rogler G, Lakatos PL, Adamina M, Ardizzone S, Buskens CJ, Sebastian S (2017). 3rd European Evidence-based Consensus on the Diagnosis and Management of Crohn’s Disease 2016: Part 2: Surgical Management and Special Situations. J Crohns Colitis.

[5] Schulze H, Esters P, Dignass A (2014). Review article: the management of Crohn’s disease and ulcerative colitis during pregnancy and lactation. Aliment Pharmacol Ther.

[6] Tracey D, Klareskog L, Sasso EH, Salfeld JG, Tak PP (2008). Tumor necrosis factor antagonist mechanisms of action: a comprehensive review. Pharmacol Ther.

[7] Hanauer SB, Feagan BG, Lichtenstein GR, Mayer LF, Schreiber S, Colombel JF, Rachmilewitz D, Wolf DC, Olson A, Bao W (2002). Maintenance infliximab for Crohn’s disease: the ACCENT I randomised trial. Lancet.

[8] Targan SR, Hanauer SB, van Deventer SJ, Mayer L, Present DH, Braakman T, DeWoody KL, Schaible TF, Rutgeerts PJ (1997). A short-term study of chimeric monoclonal antibody cA2 to tumor necrosis factor alpha for Crohn’s disease. Crohn’s Disease cA2 Study Group. N Engl J Med.

[9] Colombel JF, Sandborn WJ, Rutgeerts P, Enns R, Hanauer SB, Panaccione R, Schreiber S, Byczkowski D, Li J, Kent JD (2007). Adalimumab for maintenance of clinical response and remission in patients with Crohn’s disease: the CHARM trial. Gastroenterology.

[10] D’Haens GR, Panaccione R, Higgins PD, Vermeire S, Gassull M, Chowers Y, Hanauer SB, Herfarth H, Hommes DW, Kamm M (2011). The London Position Statement of the World Congress of Gastroenterology on Biological Therapy for IBD with the European Crohn’s and Colitis Organization: when to start, when to stop, which drug to choose, and how to predict response?. Am J Gastroenterol.

[11] Hanauer SB, Sandborn WJ, Rutgeerts P, Fedorak RN, Lukas M, MacIntosh D, Panaccione R, Wolf D, Pollack P (2006). Human anti-tumor necrosis factor monoclonal antibody (adalimumab) in Crohn’s disease: the CLASSIC-I trial. Gastroenterology.

[12] Sandborn WJ, Hanauer SB, Rutgeerts P, Fedorak RN, Lukas M, MacIntosh DG, Panaccione R, Wolf D, Kent JD, Bittle B (2007). Adalimumab for maintenance treatment of Crohn’s disease: results of the CLASSIC II trial. Gut.

[13] Stang A (2010). Critical evaluation of the Newcastle-Ottawa scale for the assessment of the quality of nonrandomized studies in meta-analyses. Eur J Epidemiol.

[14] DerSimonian R, Laird N (1986). Meta-analysis in clinical trials. Control Clin Trials.

[15] Stroup DF, Berlin JA, Morton SC, Olkin I, Williamson GD, Rennie D, Moher D, Becker BJ, Sipe TA, Thacker SB (2000). Meta-analysis of observational studies in epidemiology: a proposal for reporting. Meta-analysis Of Observational Studies in Epidemiology (MOOSE) group. JAMA.

[16] Higgins JP, Thompson SG, Deeks JJ, Altman DG (2003). Measuring inconsistency in meta-analyses. BMJ.

[17] Kiely CJ, Subramaniam K, Platten J, Pavli P (2016). Safe and effective: anti-tumour necrosis factor therapy use in pregnant patients with Crohn disease and ulcerative colitis. Intern Med J.

[18] Schnitzler F, Fidder H, Ferrante M, Ballet V, Noman M, Van Assche G, Spitz B, Hoffman I, Van Steen K, Vermeire S (2011). Outcome of pregnancy in women with inflammatory bowel disease treated with antitumor necrosis factor therapy. Inflamm Bowel Dis.

[19] Seirafi M, de Vroey B, Amiot A, Seksik P, Roblin X, Allez M, Peyrin-Biroulet L, Marteau P, Cadiot G, Laharie D (2014). Factors associated with pregnancy outcome in anti-TNF treated women with inflammatory bowel disease. Aliment Pharmacol Ther.

[20] Casanova MJ, Chaparro M, Domènech E, Barreiro-de Acosta M, Bermejo F, Iglesias E, Gomollón F, Rodrigo L, Calvet X, Esteve M (2013). Safety of thiopurines and anti-TNF-α drugs during pregnancy in patients with inflammatory bowel disease. Am J Gastroenterol.

[21] Julsgaard M, Christensen LA, Gibson PR, Gearry RB, Fallingborg J, Hvas CL, Bibby BM, Uldbjerg N, Connell WR, Rosella O (2016). Concentrations of Adalimumab and Infliximab in Mothers and Newborns, and Effects on Infection. Gastroenterology.

[22] Arsenescu R, Zhang C, Obi K, Naem M, Arsenescu V (2014). Outcome of Immunosuppressive Treatment in Pregnant IBD Patients. Am J Gastroenterol.

[23] Slama W, Roc E, Carlier P, Garayt C, Theophile H, Boyer M (2010). Pregnancy outcome in women exposed to anti tumor necrosis factor therapy. Congres de Physiologie, de Pharmacologie et de Therapeutique, Bordeaux, March 23–25, 2010.

[24] Zelinkova Z, van der Ent C, Bruin KF, van Baalen O, Vermeulen HG, Smalbraak HJ, Ouwendijk RJ, Hoek AC, van der Werf SD, Kuipers EJ (2013). Effects of discontinuing anti-tumor necrosis factor therapy during pregnancy on the course of inflammatory bowel disease and neonatal exposure. Clin Gastroenterol Hepatol.

[25] Ng SC, Shi HY, Hamidi N, Underwood FE, Tang W, Benchimol EI, Panaccione R, Ghosh S, Wu JCY, Chan FKL (2017). Worldwide incidence and prevalence of inflammatory bowel disease in the 21st century: a systematic review of population-based studies. Lancet.

[26] Rahimi R, Nikfar S, Abdollahi M (2007). Do anti-tumor necrosis factors induce response and remission in patients with acute refractory Crohn’s disease? A systematic meta-analysis of controlled clinical trials. Biomed Pharmacother.

[27] Nikfar S, Mirfazaelian H, Abdollahi M (2010). Efficacy and tolerability of immunoregulators and antibiotics in fistulizing Crohn’s disease: a systematic review and meta-analysis of placebo-controlled trials. Curr Pharm Des.

[28] Nguyen GC, Seow CH, Maxwell C, Huang V, Leung Y, Jones J, Leontiadis GI, Tse F, Mahadevan U, van der Woude CJ (2016). The Toronto Consensus Statements for the Management of Inflammatory Bowel Disease in Pregnancy. Gastroenterology.

[29] de Lima A, Zelinkova Z, Mulders AG, van der Woude CJ (2016). Preconception Care Reduces Relapse of Inflammatory Bowel Disease During Pregnancy. Clin Gastroenterol Hepatol.

[30] Selinger CP, Eaden J, Selby W, Jones DB, Katelaris P, Chapman G, McDondald C, McLaughlin J, Leong RW, Lal S (2013). Inflammatory bowel disease and pregnancy: lack of knowledge is associated with negative views. J Crohns Colitis.

[31] Lamb CA, Kennedy NA, Raine T, Hendy PA, Smith PJ, Limdi JK, Hayee B, Lomer MCE, Parkes GC, Selinger C (2019). British Society of Gastroenterology consensus guidelines on the management of inflammatory bowel disease in adults. Gut.

[32] Gomollón F, Dignass A, Annese V, Tilg H, Van Assche G, Lindsay JO, Peyrin-Biroulet L, Cullen GJ, Daperno M, Kucharzik T (2017). 3rd European Evidence-based Consensus on the Diagnosis and Management of Crohn’s Disease 2016: Part 1: Diagnosis and Medical Management. J Crohns Colitis.

[33] Côté-Daigneault J, Bouin M, Lahaie R, Colombel JF, Poitras P (2015). Biologics in inflammatory bowel disease: what are the data?. United Eur Gastroenterol J.

[34] Kane S, Lemieux N (2005). The role of breastfeeding in postpartum disease activity in women with inflammatory bowel disease. Am J Gastroenterol.

[35] Lee S, Seow CH, Adhikari K, Metcalfe A (2020). Pregnant women with IBD are more likely to be adherent to biologic therapies than other medications. Aliment Pharmacol Ther.

[36] Matro R, Martin CF, Wolf D, Shah SA, Mahadevan U (2018). Exposure Concentrations of Infants Breastfed by Women Receiving Biologic Therapies for Inflammatory Bowel Diseases and Effects of Breastfeeding on Infections and Development. Gastroenterology.

[37] Nielsen MJ, Nørgaard M, Holland-Fisher P, Christensen LA (2010). Self-reported antenatal adherence to medical treatment among pregnant women with Crohn’s disease. Aliment Pharmacol Ther.

[38] Selinger CP, Eaden J, Jones DB, Katelaris P, Chapman G, McDonald C, Smith P, Lal S, Leong RW, McLaughlin J (2013). Modifiable factors associated with nonadherence to maintenance medication for inflammatory bowel disease. Inflamm Bowel Dis.

[39] Shannahan SE, Erlich JM, Peppercorn MA (2019). Insights into the treatment of inflammatory bowel disease in pregnancy. Th Adv Gastroenterol.

[40] Singh S, Heien HC, Sangaralingham LR, Schilz SR, Kappelman MD, Shah ND, Loftus EV (2016). Comparative Effectiveness and Safety of Anti-Tumor Necrosis Factor Agents in Biologic-Naive Patients With Crohn’s Disease. Clin Gastroenterol Hepatol.

[41] Narula N, Kainz S, Petritsch W, Haas T, Feichtenschlager T, Novacek G, Eser A, Vogelsang H, Reinisch W, Papay P (2016). The efficacy and safety of either infliximab or adalimumab in 362 patients with anti-TNF-α naïve Crohn’s disease. Aliment Pharmacol Ther.

[42] Seow CH, Leung Y, Vande Casteele N, Ehteshami Afshar E, Tanyingoh D, Bindra G, Stewart MJ, Beck PL, Kaplan GG, Ghosh S (2017). The effects of pregnancy on the pharmacokinetics of infliximab and adalimumab in inflammatory bowel disease. Aliment Pharmacol Ther.

[43] Malek A, Sager R, Kuhn P, Nicolaides KH, Schneider H (1996). Evolution of maternofetal transport of immunoglobulins during human pregnancy. Am J Reprod Immunol.

[44] Mahadevan U, McConnell RA, Chambers CD (2017). Drug Safety and Risk of Adverse Outcomes for Pregnant Patients With Inflammatory Bowel Disease. Gastroenterology.

[45] Alijotas-Reig J, Esteve-Valverde E, Ferrer-Oliveras R, Llurba E, Gris JM (2017). Tumor Necrosis Factor-Alpha and Pregnancy: Focus on Biologics. An Updated and Comprehensive Review. Clin Rev Allergy Immunol.

[46] Mahadevan U, Wolf DC, Dubinsky M, Cortot A, Lee SD, Siegel CA, Ullman T, Glover S, Valentine JF, Rubin DT (2013). Placental transfer of anti-tumor necrosis factor agents in pregnant patients with inflammatory bowel disease. Clin Gastroenterol Hepatol.

[47] Kanis SL, de Lima-Karagiannis A, van der Ent C, Rizopoulos D, van der Woude CJ (2018). Anti-TNF Levels in Cord Blood at Birth are Associated with Anti-TNF Type. J Crohns Colitis.

[48] Marchioni RM, Lichtenstein GR (2013). Tumor necrosis factor-α inhibitor therapy and fetal risk: a systematic literature review. World J Gastroenterol.

[49] Deepak P, Stobaugh DJ (2014). Maternal and foetal adverse events with tumour necrosis factor-alpha inhibitors in inflammatory bowel disease. Aliment Pharmacol Ther.

[50] Lichtenstein GR, Feagan BG, Mahadevan U, Salzberg BA, Langholff W, Morgan JG, Safdi M, Nissinen R, Taillard F, Sandborn WJ (2018). Pregnancy Outcomes Reported During the 13-Year TREAT Registry: A Descriptive Report. Am J Gastroenterol.

[51] Sedger LM, McDermott MF (2014). TNF and TNF-receptors: From mediators of cell death and inflammation to therapeutic giants - past, present and future. Cytokine Growth Factor Rev..

[52] Kane SV, Acquah LA (2009). Placental transport of immunoglobulins: a clinical review for gastroenterologists who prescribe therapeutic monoclonal antibodies to women during conception and pregnancy. Am J Gastroenterol.

